# Predicting Type 1 Diabetes Candidate Genes using Human Protein-Protein Interaction Networks

**DOI:** 10.4172/jcsb.1000025

**Published:** 2009-04-01

**Authors:** Shouguo Gao, Xujing Wang

**Affiliations:** Department of Physics & the Comprehensive Diabetes Center, University of Alabama at Birmingham, 1300 University Blvd, Birmingham, AL 35294, USA

## Abstract

**Background:**

Proteins directly interacting with each other tend to have similar functions and be involved in the same cellular processes. Mutations in genes that code for them often lead to the same family of disease phenotypes. Efforts have been made to prioritize positional candidate genes for complex diseases utilize the protein-protein interaction (PPI) information. But such an approach is often considered too general to be practically useful for specific diseases.

**Results:**

In this study we investigate the efficacy of this approach in type 1 diabetes (T1D). 266 known disease genes, and 983 positional candidate genes from the 18 established linkage loci of T1D, are compiled from the T1Dbase (http://t1dbase.org). We found that the PPI network of known T1D genes has distinct topological features from others, with significantly higher number of interactions among themselves even after adjusting for their high network degrees (p<1e-5). We then define those positional candidates that are first degree PPI neighbours of the 266 known disease genes to be new candidate disease genes. This leads to a list of 68 genes for further study. Cross validation using the known disease genes as benchmark reveals that the enrichment is ~17.1 fold over random selection, and ~4 fold better than using the linkage information alone. We find that the citations of the new candidates in T1D-related publications are significantly (p<1e-7) more than random, even after excluding the co-citation with the known disease genes; they are significantly over-represented (p<1e-10) in the top 30 GO terms shared by known disease genes. Furthermore, sequence analysis reveals that they contain significantly (p<0.0004) more protein domains that are known to be relevant to T1D. These findings provide indirect validation of the newly predicted candidates.

**Conclusion:**

Our study demonstrates the potential of the PPI information in prioritizing positional candidate genes for T1D.

## Background

Dissecting the genetics of complex diseases has been challenging. Traditional linkage mapping approaches, developed based on single disease gene concept, have been less powerful due to locus heterogeneity and epistasis (Glazier et al., 2002; Botstein and Risch, 2003). According to T1Dbase (http://t1dbase.org), a public website and database that supports the type 1 diabetes (T1D) research community (Hulbert et al., 2007), 18 chromosome regions have shown linkage to T1D (at least in some populations), and are believed to harbour disease genes. They vary in length and gene count; most contain numerous, up to 277 (the IDDM4 locus), genes. For most loci the sequence variation responsible for the linkage has not been identified (Atkinson, 2005).

This problem is not unique to T1D, most complex human diseases are facing the same difficulty. A number of bioinformatics/integrative approaches have been developed to prioritize and narrow down the positional candidate gene list obtained from linkage peaks, by bringing in other types of data of the genes, including expression patterns, ontological annotations, and text mining of PubMed abstracts, etc. For example, several studies have utilized gene expression profiles in relevant tissues and/or the eQTL information of the gene expressions (Zhu et al., 2004; Cervino et al., 2005; Chesler et al., 2005; Schadt et al., 2005; Gandhi et al., 2006), including in the study of T1D (Eaves et al., 2002). Recently, approaches to prioritize candidates based on their functional relatedness to the known disease genes are explored. This is based on the concept that complex human diseases are caused by multiple genes. Since they together lead to the same or similar disease phenotypes, the genes are likely to be related functionally. Such functional relatedness can be inferred from their functional annotation, co-expression pattern, and protein-protein interaction (PPI) networks, etc. Indeed, analysis of known disease genes revealed that those of the same diseases tend to have higher and synchronized expressions as a group, and to interact (PPI) with each other (Xu and Li, 2006). Therefore these characteristics can be utilized in prioritizing positional candidates and novel disease gene discovery. For example, Frankee et al. proposed to rank genes in candidate regions by their relatedness to candidates in other regions, which was evaluated according to their sharing in pathway and GO (Gene Ontology) annotations, microarray co-expression, and PPI (Franke et al., 2006). Bergholdt et al. (2007) used PPI to identify network modules that contained significant enrichment of proteins from interacting regions, and hence novel candidate genes for T1D. Of these characteristics, direct PPI is one of the strongest manifestations of a functional relation between genes. Recent studies showed that mutations to interacting proteins can lead to similar disease phenotypes (Lage et al., 2007; Sieberts and Schadt, 2007). High throughput analysis of all OMIM (Online Mendelian Inheritance in Man, http://www.ncbi.nlm.nih.gov/entr ez/quer **y**.fcgi?db=OMIM) human diseases also indicated the potential of utilizing PPI information alone to prioritize disease gene candidates (Oti et al., 2006).

Such integrative genomics approaches, though show promise theoretically in a general sense, are still often considered not practically useful for specific diseases (Oti and Brunner, 2007). In this study, we will first examine the PPI network structure of the known T1D genes. Based on the results, we design an algorithm to prioritize the positional candidates according to their PPI with the known T1D genes. It leads to the identification of 68 new candidates. We examine the likelihood of their involvement in T1D from several aspects including their functional annotation, independent citation in T1D-related publications, and protein sequence domain characteristics. Our study differ from previous T1D work by others (Bergholdt et al., 2007) in that we take all the known functional and positional candidates (according to T1DBase) as a starting point, rather than limit to only the positive predictions from the recent genome wide association studies. Further, we offer a comprehensive evaluation of our novel predictions.

## Methods

### T1D Genetic Data

The following data were downloaded from T1Dbase: the complete list of 266^1^ known functional candidate genes of T1D (which will be termed known T1D genes in this study); the 983 positional candidate genes from the 18 known T1D linkage regions; and information of T1Drelated and all Entrez Gene publications. T1Dbase compiled the list of known functional candidate genes for T1D from the Genetic Association Database (http://geneticassociationdb.nih.gov, genes shown association to human diseases were curated from genetic studies reported in published scientific papers), and from genes deemed of interest to T1D by the Wellcome Trust Diabetes and Inflammation Laboratory. The 18 linkage regions were compiled from published genome scan duties. More detail can be found from its website http://t1dbase.org.

### Candidate Gene Prediction

PPI annotation was downloaded from the Human Protein Reference Database (HPRD http://www.hprd.org/). Additionally, two high throughput PPI datasets by Rual, et al. (2005) and Stelzl, et al (2005) were obtained from the supplementary material of their papers, and were combined. The pooled dataset is referred to as HT (high throughput) in this study. The significance of the PPI enrichment among the T1D genes was evaluated using the bootstrapping method. For each of 20 times, we randomly selected the same number of genes from the HPRD or the HT dataset, and determined the PPI among them. The results were then used to determine the PPI statistics for a random list of genes.

The first-degree (level-1) PPI neighbours of all known disease genes were first determined and called baited genes. The algorithm then went through all 983 positional candidates, and identified those that are baited genes to be new candidate disease genes. The number of independent baits (known T1D genes) for each baited gene was also determined. The latter was in turn used to investigate if a gene with more disease gene partners is more likely to be also a disease gene. This could lead to further prioritization of the predicted candidates.

Functional analysis of known and predicted T1D genes were carried out using GOStat (Beissbarth and Speed, 2004). Compared with other ontological analysis tools, it has the advantage that parent-child relationship between the GO terms are considered (Beissbarth and Speed, 2004). The protein domain information was retrieved from InterPro (http://www.ebi.ac.uk/interpro/), and Fisher's test was used to examine domains overrepresented in known and predicted T1D genes (Mulder et al., 2005).

## Results

### Topological Features of the PPI Network of known T1D Disease Genes

**Figure 1:**
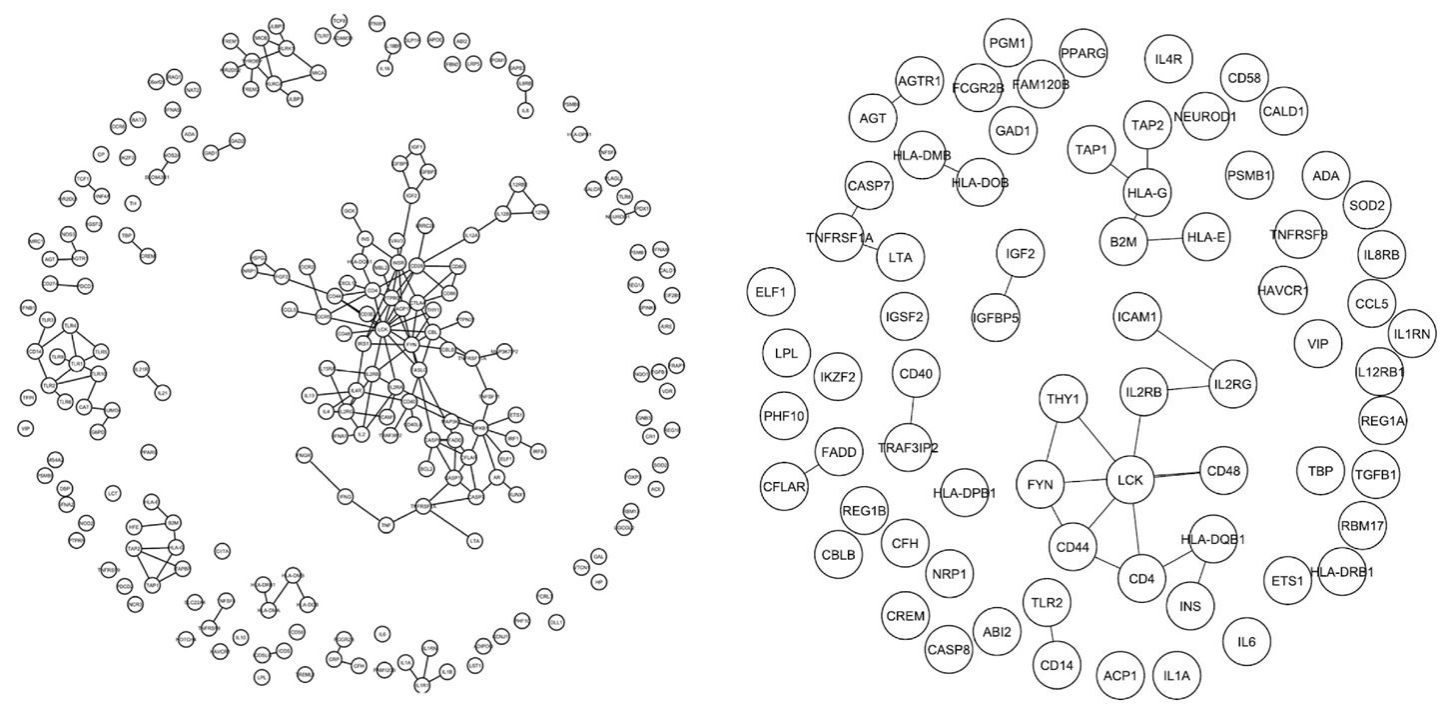
PPI networks of T1D disease genes according to HPRD (left) and HT (right).

To avoid any potential bias toward well studies genes (whose interaction with other genes are better characterized) (Oti and Brunner, 2007; Ideker and Sharan, 2008), we initially examined the PPI networks using information both from the HPRD annotation, and from the 2 HT data sets (Rual et al., 2005; Stelzl et al., 2005). Figure 1 presents the results. We found that the T1D genes interact with each other significantly more often than randomly selected gene sets. Of all 20152 known human genes (according to NCBI's Gene database), 9222 are annotated in HPRD, and 4157 in HT. For the 266 known T1D genes, 222 are annotated in HPRD, and 75 in HT. There are a total of 34398 edges (in network's language, each node represents one protein molecule, and an edge between two nodes means the two molecules interact with each other) among the 9222 proteins in HPRD, and 9277 edges among the 4157 proteins in HT. The numbers for the T1D genes are 169 in HPRD, and 25 in HT, respectively. In contrast, bootstrapping yields only 21.1±4.2 and 3.7±2.3 interactions for a random gene set of the same sizes. These are 8.0 and 6.8 fold enrichment, respectively. The results from HPRD and HT are comparable, and we do not observe any noticeable bias in the HPRD dataset. In the rest of this study, we used HPRD only as it contains more comprehensive information of PPI.

It has been found that proteins of disease genes often possess higher network degrees (i.e. number of interactions with other proteins) than randomly selected genes (Tu et al., 2006; Xu and Li, 2006). We found that this is indeed true for the T1D genes (p<0.001, Kolmogorov-Smirnov test, or, KS-test). This raises a question that whether the enrichment was brought in by the higher degrees of the known disease genes? To answer this question we bootstrapped random genes with the same degree distribution. Significant enrichment was still observed (p<1e-5), suggesting independent contributions from other sources, likely their close functional relatedness.

**Figure 2:**
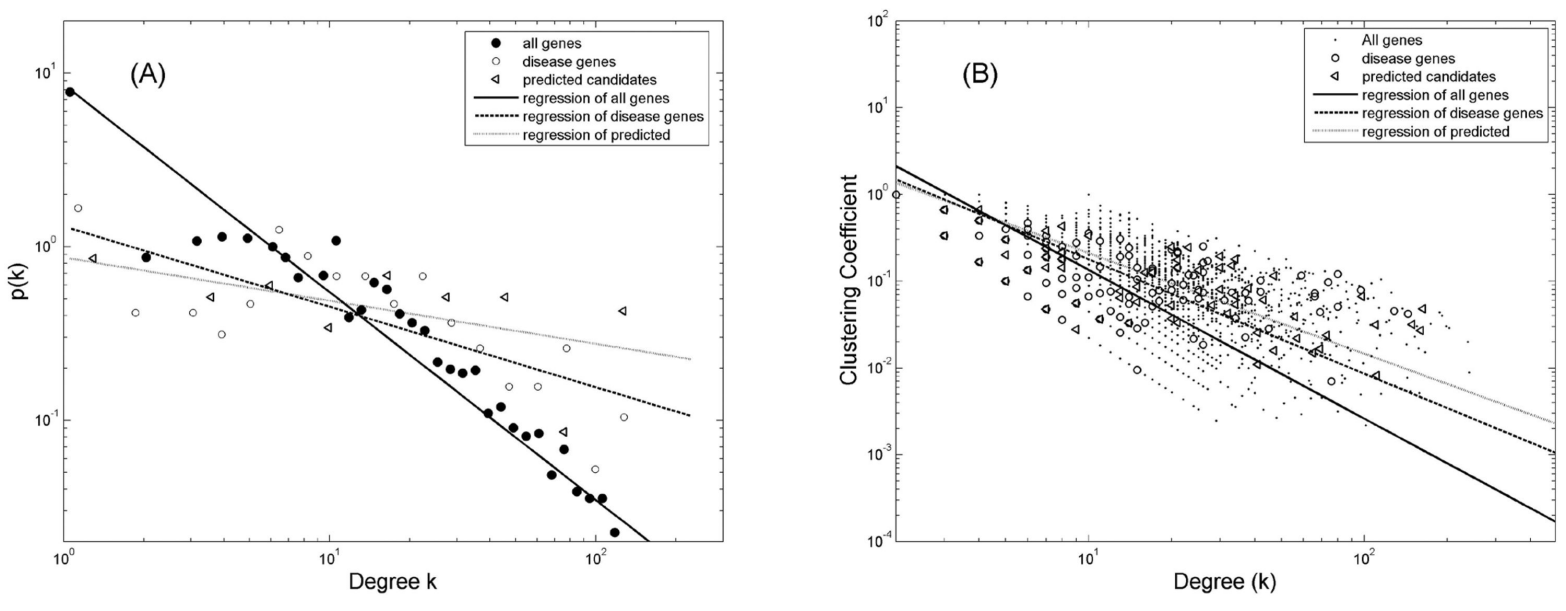
The topological features of the T1D disease genes in the PPI network are distinct from the other genes. (A) The degree distribution of all proteins follows a power law (r~0.98, p<0.001), with *p*(*k*)~*k*^−λ^ , λ ~1.35, indicating the PPI network is scale free. The distribution for the candidate genes clearly deviate from the power law, skewed significantly toward higher degrees. (B) The clustering coefficient (CC) is plotted against degree *k*. There is a linear decline in CC with increasing *k*, suggesting that the network is modular. The distribution of the disease genes again deviate from random genes, with more interactions among their level-1 neighbours.

Genetic networks have been found to be different from random networks in structure. For example, they often exhibit small-world and scale-free properties (Barabasi and Oltvai, 2004). Therefore merely comparing the average network behaviour may not be adequate. For this reason, we also examined the topological properties of the disease gene PPI networks. We find that the degrees of all proteins follow a power law w *p*(*k*)~*k*^−λ^ , with λ ~1.35, r~0.98, and p<0.001 (figure 2A), where *p*(*k*) is the probability density. This indicates the PPI network is scale free. The distribution for the 222 disease genes clearly deviate from the power law, skewed significantly toward higher degrees, suggesting that disease genes tend to have more interaction partners. We also examined the clustering coefficient (CC) and its dependence on degree k. CC measures how first degree neighbours of the same node interact with each other, namely, the cliquiness. Again a power law decline with increasing k is evident (figure 2B, r~0.70, p<0.01), suggesting that the network is of modular structure (Barabasi and Oltvai, 2004). Here the known disease genes once more deviate from the average behaviour of all genes in the genome, skewed toward higher CC at the same degree k, with a much shallower slope (0.67 versus 0.93, p~0.00014). This implies that the disease genes likely form subnetwork modules with much higher internal interactions than with genes outside the module.

These characteristics of disease genes are not unique to T1D, they in fact emulate the results of similar studies of other diseases, where it was found that disease genes tend to have larger degrees, more likely to interact with other disease genes, and share more common neighbours (Tu et al., 2006; Xu and Li, 2006). These results provide the conceptual basis for candidate gene prediction utilizing PPI with known disease genes.

### Cross Validation of the Candidate Gene Prediction Algorithm

**Figure 3:**
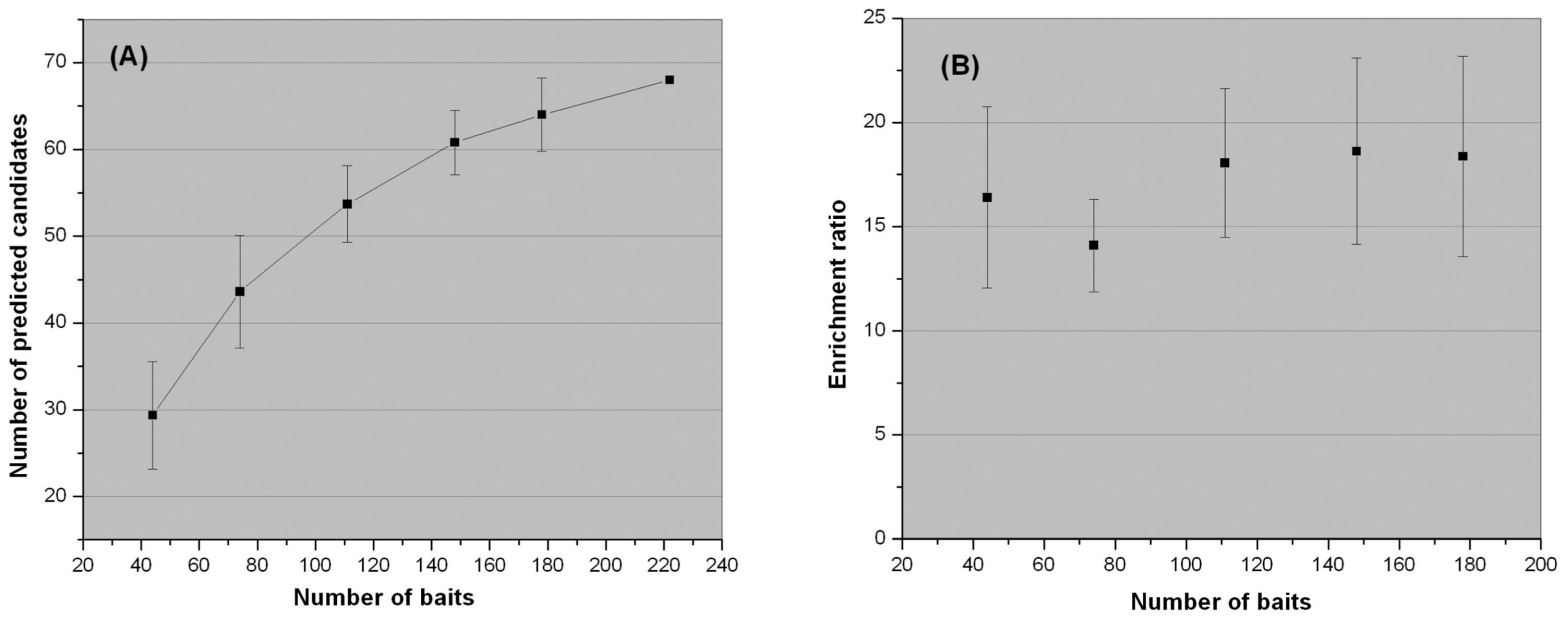
The size effect of the bait set. (A) Number of predicted disease genes increases with number of baits. (B) The efficiency of the disease gene prediction algorithm, as judged by the odds ratio of known disease gene being recovered, does not depend on the size of bait set.

We first evaluated the performance of the disease gene prediction algorithm using the known T1D genes as bench marks. In more detail, each time we randomly select *f* fraction of known T1D genes as baits, and tested how many of the remaining 1-*f* fraction were predicted. We tested for 6 different *f* values: 1/5, 1/3, 1/2, 2/3, 4/5 and 1, and for each *f* value (except *f*=1, which was only used to calculate the number of predicted genes, but not for cross validation as no testing set) we repeated 20 times. Figure 3 summarizes the results. Evidently the number of predicted genes increases with the number of baits (figure 3A). Interestingly, the trend seems to slow down as the bait number increases. This could be due to the limitations of our current knowledge of PPI (incompleteness and quality issues, for example), it may also suggest that total number of T1D disease genes is limited. Further investigation of this phenomenon is needed when we have a better understanding of PPI and T1D disease biology. The efficiency to recover the known disease genes, defined as the odds of disease gene enrichment in predicted candidates over random, seems to be affected little by the number of baits, as shown in figure 3B. The high enrichment ratios, at ~17.1 (14.1-18.6) fold suggest that our baiting algorithm can recover the known disease genes well.

How much improvement in predictive power did the addition of PPI information bring in? The 18 known T1D linkage loci together offer 983 positional candidates. Using the known disease genes as bench marks, 59 of the 266 T1D genes are within the linkage regions, thus the linkage data by itself lead to a 4.5-fold enrichment (p<1e-17, Fisher's exact test). If we restrict to only the 9222 genes annotated in HPRD, 487 are within the linkage region. For the 222 disease genes annotated in HPRD, 52 are within the linkage regions. The enrichment by linkage information alone is similar at ~4.4 fold (p<1e-15). Therefore, the PPI with known disease genes brought in an additional ~4 fold of enrichment.

### Predicted New Candidates

**Figure 4:**
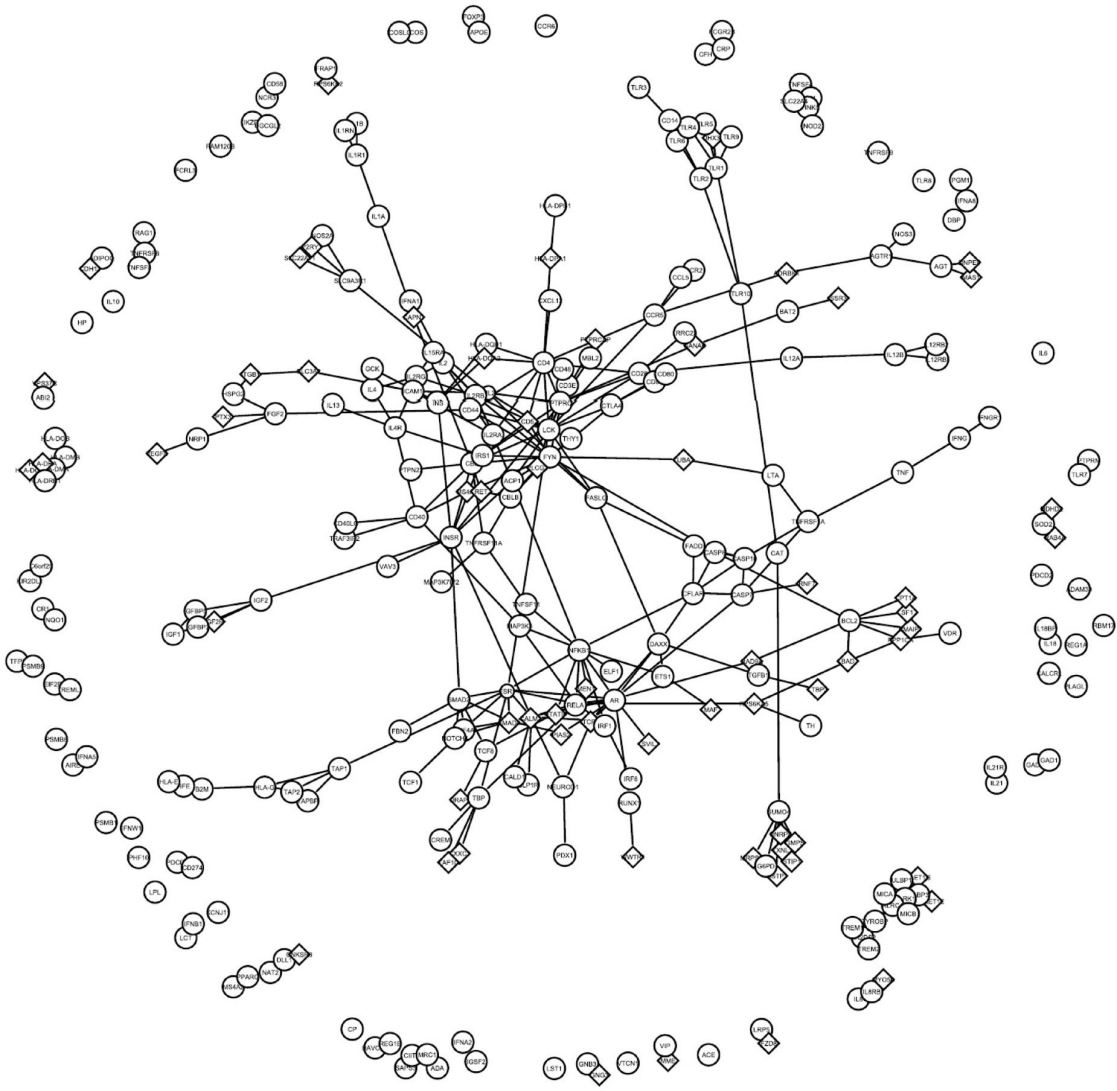
The PPI network of known (circle) and predicted disease genes (diamond).

Using all 222 T1D genes (annotated in HPRD) as baits, we arrived at a list of 68 predicated new candidates, given in table 1. None of these has been previously associated to T1D according to T1Dbase. Figure 4 depicts the interactions between all known and predicted T1D genes.

### Network Properties

The network properties of the predicted genes are significantly different from the average HPRD annotated genes, and are much closer to the known T1D genes. The number of interactions among themselves is significantly higher than random (p<0.00001). In figures 2A and 2B, we have also included plots of the predicted candidates. Evidently they cluster with the known T1D genes, concentrate more to the high-degree end (Figure 2A), and share more first degree neighbours than random (figure 2B).

### Functional Properties

In table 2 we listed the top 30 GO molecular function categories shared among the 222 known disease genes (p<1e-22), and their statistics in the 68 new candidates. These categories clearly indicate an involvement of immunity, which is consistent with T1D being an autoimmune disease. All categories have enhancement ratio above 1, except for the 4 with very low (0 or 1) representations in the 68 predicted genes, which are sensitive to random effect. 14 have enhancement ratio above 2. Putting all GO terms together, they are significantly (p<1.3e-10) over-represented in the new candidates.

### Protein Sequence Analysis

The function of a protein is determined by its shape and primary structure (Mulder and Apweiler, 2008). InterPro is an integrated database of protein families, domains and functional sites. We examined the protein motifs that are over-represented in the known and predicted disease genes. Listed in table 3 are the top 10 (Fisher's exact test, p<1e-16) motifs shared among the known disease genes. 6 of them are also over represented in the 68 new candidates. For the remaining 4, the expected number of genes that share the motif (i.e. (# of the 9222 that share the motif)/(9222/68)) is far less than 1 (all below 0.25), therefore we do not have enough statistical power to determine if they are over represented or not. Taking the results from the 6 informative motifs together, it suggests that the predicted genes participate in similar biological processes as the known T1D genes. Again immune related sequence features are overrepresented in both the known and predicted genes, consistent with the fact that T1D is an autoimmune disease.

### Literature Support

To investigate the potential T1D relevance of the new predictions, we further examined the literature citation of both known and predicted disease genes. For each gene we obtained the total number of PubMed citations and the fraction that are T1D-related (according to T1Dbase). For the predicted genes, one may argue that their appearance in T1D publications could be a result of their interactions with the known disease genes, as interacting genes often appear in the same publications. To address this issue, we excluded from the analysis of the predicted genes all PubMed records that have cited the known T1D genes.

**Figure 5:**
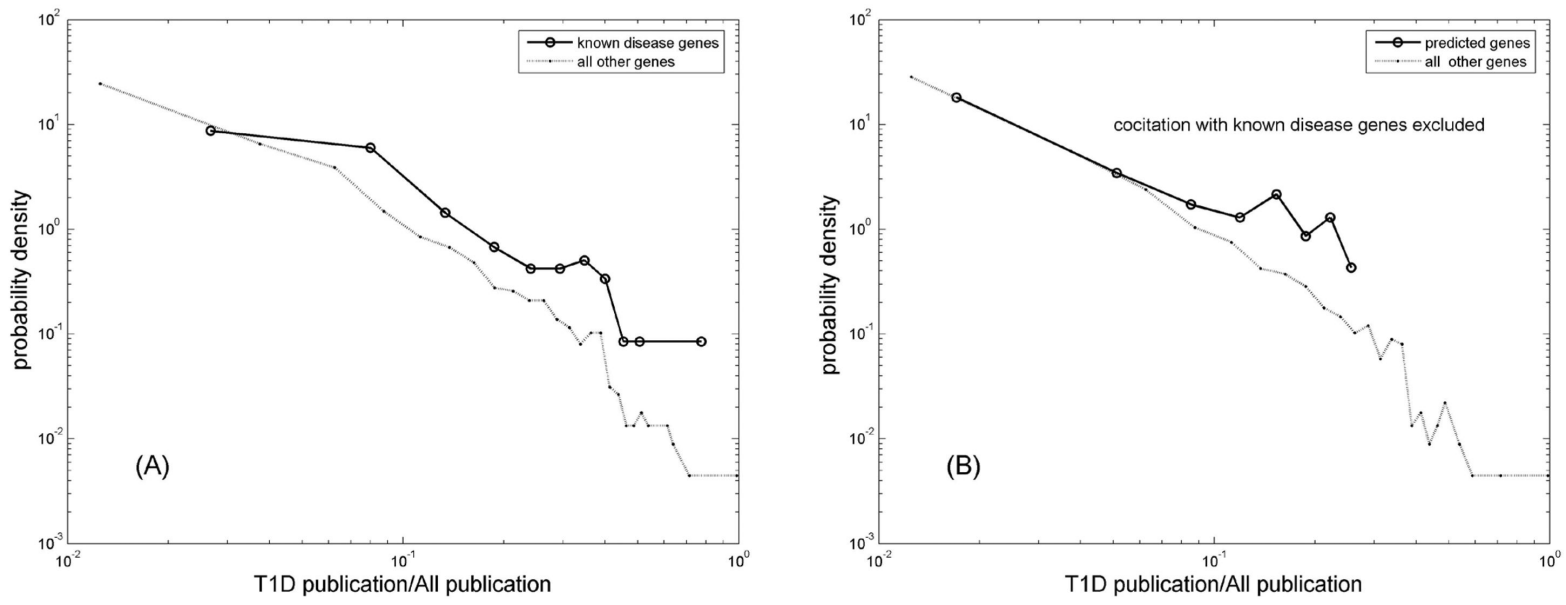
The probability density distribution of normalized T1D citation. Both known (A) and predicted disease genes (B) are cited significantly (p<1e-33, and p<1e-5, respectively, KS-test) more often in T1D-related publications than random genes. In the analysis of predicted, cocitations with known disease genes were excluded.

We found that out of the 68 new candidates 13 (~20%) are cited significantly more often than random in T1D publication at p<0.05 (Fisher's exact test), as compared to only ~6.9% of the HPRD genes. This is a ~3-fold enrichment. As a group members of the 68 list are significantly (p<1e-7) more likely to appear in T1D-related publications than members of a random set of 68 genes. Figure 5 presents a more quantitative evaluation, by plotting the probability density distribution of the fraction of T1D-related citations. Interestingly, the citation seems to also follow the power law approximately. As expected, the distribution for known disease genes is significantly skewed toward higher T1D-related citations (p<1e-033, KS-test, figure 5A). Of interest is the fact that even after removing the co-citations with the known T1D genes, the newly predicted disease genes are also cited more often in T1D literature (p<1e-5, KS-test, figure 5B). These results provide a strong indirect evidence of their potential involvement in T1D.

### Number of Interactions with the known Disease Genes, Possibilities to Prioritize the Predicted Genes?

Out of the 68 novel candidates, more than a third (24) interact with at least two known disease genes, and about a sixth (12) interact with at least three. This raises the question whether interacting with more disease genes means higher likelihood of also being a disease gene, and if such information can be used to further prioritize the prediction. This is intuitive as subsets of genes having much more interactions with each other than with others are likely to be from a same functional network module, and consequently to be involved in the same physiological processes and disease phenotypes.

**Figure 6:**
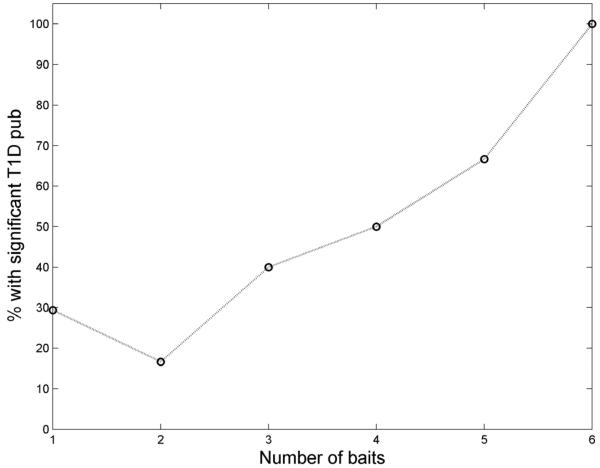
Candidates predicted by more baits are more likely to be cited in T1D-related publications.

We found that the Pearson correlation between the number of baits and the significance of T1D citation (−log10(p), *excluding co-citation with the known disease genes*) was ~0.45. In figure 6 the fraction of genes with significant T1D-related citations was plotted against number of baits. A loose cut-off, p<0.2, was used due to the small number of predicted genes. A positive monotonic trend is evident. We also used KS-test to quantitatively evaluate this question. Using 2, 3 and 4 baits as a cut-off we divided the 68 genes into groups of low and high number of baits and examined the significance distribution in each group. We found that with any cut-off the two groups are different with p<0.032 (2), p<0.019 (3), and p<0.05 (4), respectively. These all suggest that the number of interactions with known disease genes is likely an indicator of the candidate's likelihood being a disease gene.

**Figure 7:**
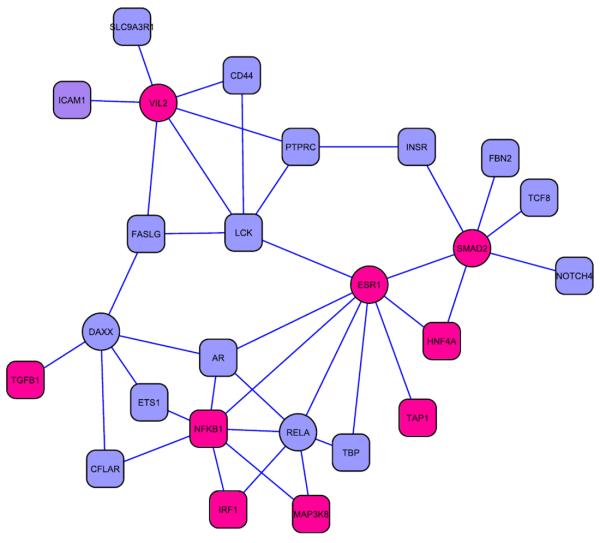
PPI network of top 5 predictions (ellipse) and their corresponding baits (round rectangle). Bright magenta nodes represent genes with significant citation in T1D-related publications (p<0.01).

Figure 7 shows the PPI network of the top 5 candidates in terms of number of baits. On the top are ESR1 and VIL2, each with 6 baits (table 1). Interestingly, they are also among the top in terms of independent citations in T1D-related publications and network degrees. ESR1, or estrogen receptor 1, has been cited in 139 (124, after removing co-citation with known disease genes) T1D-related publications, which ranked number 1 (1) out of the 68 candidates; the number for VIL2 is 30 (29), ranked number 8 (7). The odds ratios to random genes are all greater than 1, at 9.6 for VIL2 and 6.2 for ESR1, with p~8.2e-19 and p~4.2e-9 (Fisher's test), respectively. Both have abundant interactions with other proteins, with k=163, #1 of the 68 for ESR1; and k=43, #11 for VIL2. These are within the top 2% of all genes, and both can be considered hubs.

ESR1 is within the IDDM5 locus located at 6q25, and has been purported to be responsible for the linkage (Pietropaolo and Le Roith, 2001). IDDM5 is one of the few susceptibility regions that have been replicated in multiple studies (Pociot and McDermott, 2002). In addition, it is a major disease gene for type 2 diabetes, and is strongly associated with obesity and lipid metabolism. VIL2 (also known as EZR, or ezrin), is also located in IDDM5. Compared with ESR1, it is a much less studied gene. It encodes a cytoplasmic peripheral membrane protein that plays a key role in cell surface structure adhesion, migration and organization. It has been implicated in various human cancers. Its role in T1D pathogenesis is still not clear, though multiple studies have linked it in the progression and complication of diabetes (Goh and Cooper, 2008).

The next on the list are three genes that each interacts with 5 known T1D genes: SMAD2, RELA and DAXX. The number of independent citations in T1D-related publications are 52 (#3, p<4.2e-18), 35 (#14, p~0.060), 6 (#21, p~0.42), respectively. They are all highly connected genes, degrees all in the top 5% of the 9220 HPRD proteins, with k=160 (#2 of the 68), k=98 (#5), and k=34 (#14) respectively.

SMAD2 is a member of the SMAD family. Proteins of this family are signal transducers and transcriptional modulators that mediate multiple signaling pathways. SMAD2 mediates the signal of the transforming growth factor (TGF)-beta, and thus regulates multiple cellular processes, such as cell proliferation, apoptosis, and differentiation. TGF-beta plays a central role in activation of inflammation, and in the regulation of anti-islet CD8+ T cells by the CD4+CD25+ T regulatory cells during T1D (Green et al., 2003). The secretion of TGF-beta in recent onset T1D has been observed to be elevated (Stechova et al., 2007). RELA is also known as p65. Its protein is involved in the forming of the NFêB complex. NFêB1 or NFêB2 is bound to REL, RELA, or RELB to form the NFêB complex. The NFêB1 (p50)/ RELA (p65) heterodimer is the most abundant form of the complex. NFêB activation has been implicated in the protection of target cells against apoptosis by a variety of death effectors, including cytokine mediated β-cell death (Chang et al., 2003). DAXX, death-associated protein 6, is in the extended MHC region (IDDM1). There is evidence of its involvement in the T1D disease pathways in patients displaying intermediate risk DQ-DR haplotypes (van der Slik et al., 2007). It binds the receptor of TGF-beta and modulate the TGF-beta apoptotic-signalling pathway (Perlman et al., 2001). It physically interacts with the insulin-sensitive glucose transporter, GLUT4 (Lalioti et al., 2002).

## Discussion

Increasing evidence suggest that interacting proteins often share similar function, and participate in the same biological pathways and processes (Oti and Brunner, 2007). Therefore mutations in genes coding for them could lead to similar disease phenotypes. These facts indicated that PPI information alone may offer a simple, efficient means to annotate protein functions and to prioritize candidate genes for complex human diseases (Oti et al., 2006). In this study we carried out a comprehensive PPI network analysis of the known T1D disease genes. We found that they cluster in the high degree region, more likely to interact with each other, and share more common interaction partners. We then examined the potential of using PPI with known disease genes in prioritizing the positional candidates of T1D.Among the 983 genes within the 18 T1D linkage loci, 68 are first degree PPI neighbours of the known T1D genes, which we defined as the new candidate disease genes. Cross validation indicates that the approach is ~17.1 fold better than random selection to recover disease genes. Examination of the new candidates revealed that they share with the know disease genes a significant amount of GO categories and protein sequence motifs that are known to be important to autoimmunity. Furthermore, they are cited significantly more often in T1D-related publications, independent from their co-citation with the known disease genes. These all provide indirect support for their candidacy.

Here we only used the direct interaction relationship among genes. More sophisticated features, such as topological overlap (Zhang and Horvath, 2005), average distance to disease genes, positive topological coefficient (Xu and Li, 2006), are worthy of consideration in future research. Our analysis of the novel candidates rely heavily on the present protein and gene annotation databases, and the available literature report of studies related to T1D. Therefore it is likely limited by the quality of the PPI and linkage data, and the current understanding of the T1D aetiology. Not all the 266 known T1D genes can be consistently replicated in different populations, nor the 18 linkage regions (Atkinson, 2005). The recently published genome wide association studies (GWAS) only confirmed a few of the previously identified regions whilst offering evidence for yet several new regions (Hakonarson et al., 2007; The Wellcome Trust Case Control Consortium, 2007; Todd et al., 2007). There is a tendency presently to consider the GWAS results being the ultimate verdict and view previous findings that not confirmed by GWAS as false positives. If so, most of the 266 genes and the 18 regions could be false positives. It is rather intriguing then the new candidates predicted by our algorithm show strong evidence in their potential involvement in T1D, especially the independent citation in T1Drelated publications. Here we would like to emphasize that the GWAS studies are only adequately powered to detect very common alleles unless they greatly increase disease risk, and explains little the genetic variation of disease. The intricacy of complex human diseases itself further compound the interpretation of the results from genetic studies. Population difference, disease heterogeneity, the genetic mechanism of the disease including alleles with small effect sizes, epi-static interaction, epigenetic inheritance, copy number variation, etc, all raise the question of how much reliance one should give to a individual type of genetic data obtained from a certain population, including the GWAS. However, we believe that by taking an integrative approach, and examine the convergent predictions, the noise and consequently the false positives will be reduced, and true signals will be amplified. Therefore, before our understanding of the disease aetiology improves, it is better to be inclusive at the beginning of an integrative approach.

68 candidates may still be too many for association or functional studies. Further prioritization is needed. The results of this study suggest that the topological features in the PPI network with known disease genes, the functional and sequence information, and the literature citation can provide further discrimination of the predicated candidates. For example, it is possible to rank them according to their position and degree in the PPI network, degree of interaction with known disease genes, citation by T1D-related publications, protein sequences motif, as well as expression pattern, and gene ontology. A composite metric could be defined for candidates based on these properties to describe their likelihood of being true disease genes. These properties are not necessarily all independent. As an example, figure 6 illustrates the potential confounding between number of interacting disease genes and the number of citations in T1Drelated publications. Therefore, when designing such composite measures, sophisticated approaches such as the Bayesian method, which can handle non-independent factors, are needed. We are investigating these issues in a separate study (manuscript in preparation).

In this study we focused on prioritizing positional candidate genes within the linkage loci. By its nature, the approach can be applied to candidate genes obtained by other means. With the advancement of the human genome and the HapMap projects, emerging technological advances make the GWAS a reality for many laboratories to identify genetic variants that contribute to common diseases (Hirschhorn and Daly, 2005; Wang et al., 2005). While GWAS has the potential to catch all disease genes, sample size and power issues, among others, still limit its ability to obtain a complete picture of the genetic risk; or to identify genes that in combination cause disease predisposition, while each on its own only contribute moderately to the risk. GWAS typically produces a large number of potential candidate genes. Normally, only markers with extremely low p-value (usually <~1e-7) are retrieved because of the power and multiple testing issue. Lowering the threshold will be plagued with false positives, though it is believed that a region immediate below the threshold p value harbours many true disease genes (The Wellcome Trust Case Control Consortium, 2007). These regions need to be investigated to fully dissect the genetics of complex diseases. The significance of novel candidates can be investigated further, by including the GWAS results in the definition of the composite likelihood measures of the prioritization scheme. On the other hand, information obtained from other approaches such as the PPI networks, can in turn also help the analysis of the GWAS data. An analytical prioritization scheme that brings in other evidence potentially will allow one to narrow down the number of statistical tests to be performed, and to identify disease genes from the sub optimal p-value regions.

## Figures and Tables

**Table 1:** List of the 68 predicted disease genes.

Gene ID	Gene Name	Linkage locus*	# of baits	PubMed Citation	Citation in T1D- related publications		T1D citation, excluding co-citation with baits	
					#	p	#	p
2099	ESR1	IDDM5	6	783	139	1.49E-27	124	8.3E-19
7430	VIL2	IDDM5	6	117	30	2.45E-10	29	4.2E-09
1616	DAXX	IDDM1	5	85	7	0.22	6	0.42
4087	SMAD2	IDDM6	5	172	82	7.2E-41	52	4.2E-18
5970	RELA	IDDM4	5	429	36	0.019	35	0.060
801	CALM1	IDDM11	4	189	18	0.030	16	0.13
921	CD5	IDDM4	4	123	2	0.99	1	1
3118	HLA-DQA2	IDDM1	3	13	0	1	0	1
3122	HLA-DRA	IDDM1	3	123	36	1.6E-13	31	8.9E-10
4089	SMAD4	IDDM6	3	203	82	1.0E-36	60	3.1E-20
5336	PLCG2	UN16	3	91	2	0.96	2	0.97
10524	HTATIP	IDDM4	3	65	4	0.51	3	0.75
156	ADRBK1	IDDM4	2	104	34	5.5E-14	32	6.5E-12
823	CAPN1	IDDM4	2	101	3	0.92	3	0.94
931	MS4A1	IDDM4	2	59	0	1	0	1
3113	HLA-DPA1	IDDM1	2	31	1	0.83	0	1
5499	PPP1CA	IDDM4	2	82	3	0.84	1	0.99
5883	RAD9A	IDDM4	2	60	2	0.85	2	0.88
5979	RET	IDDM10	2	471	12	1	7	1
6925	TCF4	IDDM6	2	71	10	10	3	0.80
7277	TUBA1	IDDM13	2	68	13	0.00036	10	0.013
10589	DRAP1	IDDM4	2	25	0	1	0	1
23193	GANAB	IDDM4	2	25	0	1	0	1
353091	RAET1G	IDDM5	2	3	0	1	0	1
572	BAD	IDDM4	1	138	9	0.39	4	0.96
1012	CDH13	UN16	1	50	3	0.55	1	0.95
1374	CPT1A	IDDM4	1	60	4	0.45	4	0.50
2785	GNG3	IDDM4	1	34	0	1	0	1
2950	GSTP1	IDDM4	1	177	9	0.67	6	0.95
3111	HLA-DOA	IDDM1	1	31	1	0.83	0	1
3185	HNRPF	IDDM10	1	35	0	1	0	1
3482	IGF2R	IDDM5	1	183	3	1	2	1
3688	ITGB1	IDDM10	1	494	89	1.2E-18	83	2.3E-14
4054	LTBP3	IDDM4	1	36	7	0.0074	5	0.083
4094	MAF	UN16	1	70	9	0.025	7	0.15
4142	MAS1	IDDM5	1	53	0	1	0	1
4221	MEN1	IDDM4	1	107	17	0.00035	16	0.0018
4311	MME	IDDM9	1	133	9	0.35	9	0.43
4645	MYO5B	IDDM6	1	31	0	1	0	1
5028	P2RY1	IDDM9	1	74	16	2.2E-05	13	0.0013
5366	PMAIP1	IDDM6	1	37	0	1	0	1
5790	PTPRCAP	IDDM4	1	32	0	1	0	1
5806	PTX3	IDDM9	1	51	1	0.94	1	0.95
5867	RAB4A	UN1	1	55	1	0.95	1	0.96
6199	RPS6KB2	IDDM4	1	34	2	0.58	1	0.87
6520	SLC3A2	IDDM4	1	94	10	0.052	7	0.36
6747	SSR3	IDDM9	1	16	8	2.3E-05	6	0.0012
6840	SVIL	IDDM10	1	21	3	0.14	2	0.38
7423	VEGFB	IDDM4	1	49	0	1	0	1
7536	SF1	IDDM4	1	41	8	0.00429	7	0.019
8325	FZD8	IDDM10	1	27	6	0.0075	5	0.034
8833	GMPS	IDDM9	1	18	0	1	0	1
9013	TAF1C	UN16	1	23	0	1	0	1
9063	PIAS2	IDDM6	1	56	5	0.23	3	0.66
9252	RPS6KA5	IDDM11	1	53	4	0.36	2	0.83
9352	TXNL1	IDDM6	1	20	0	1	0	1
9616	RNF7	IDDM9	1	31	1	0.83	1	0.84
10963	STIP1	IDDM4	1	48	0	1	0	1
23549	DNPEP	IDDM13	1	17	0	1	0	1
25937	WWTR1	IDDM9	1	18	1	0.65	0	0.65
30827	CXXC1	IDDM6	1	23	1	0.73	0	0.73
55048	VPS37C	IDDM4	1	14	0	1	0	1
55867	SLC22A11	IDDM4	1	15	0	1	0	1
56945	MRPS22	IDDM9	1	19	0	1	0	1
84064	HDHD2	IDDM6	1	15	0	1	0	1
135250	RAET1E	IDDM5	1	19	0	1	0	1
154043	CNKSR3	IDDM5	1	10	2	0.13	0	0.13
170506	DHX36	IDDM9	1	20	0	1	0	1

* most loci were named IDDM#, where IDDM stands for Insulin Dependent Diabetes Mellitus, another name for type 1 diabetes.

**Table 2:** The top 30 GO categories shared by the 266 known T1D genes, and their presentation in the 68 predicted disease genes.

GO term	# of genes in the 68 list	# in HPRD	Enrichment rtio	p
|1|GO:0002376|immune system process|	9	640	2.2834	0.06
|2|GO:0006955|immune response|	5	480	1.7634	0.30
|3|GO:0006952|defense response|	4	397	1.5896	0.35
|4|GO:0048522|positive regulation of cellular process|	16	748	3.2124	0.0005
|5|GO:0048518|positive regulation of biological process|	16	835	2.874	0.0015
|6|GO:0009607|response to biotic stimulus|	2	166	1.871	0.35
|7|GO:0005102|receptor binding|	4	566	1.0546	0.60
|8|GO:0031325|positive regulation of cellular metabolic process|	8	313	3.9479	0.0037
|9|GO:0009893|positive regulation of metabolic process|	8	329	3.7422	0.0049
|10|GO:0005615|extracellular space|	0	356	0	1
|11|GO:0042127|regulation of cell proliferation|	3	359	1.275	0.50
|12|GO:0009611|response to wounding|	3	345	1.3251	0.48
|13|GO:0009605|response to external stimulus|	4	470	1.2709	0.47
|14|GO:0008283|cell proliferation|	5	596	1.236	0.46
|15|GO:0051707|response to other organism|	1	113	1.4804	0.57
|16|GO:0045321|leukocyte activation|	4	177	3.9334	0.048
|17|GO:0001775|cell activation|	4	198	3.4324	0.067
|18|GO:0044421|extracellular region part|	0	507	0	1
|19|GO:0046649|lymphocyte activation|	4	159	4.3458	0.036
|20|GO:0005886|plasma membrane|	18	1402	1.8357	1.8357
|21|GO:0044459|plasma membrane part|	17	1146	2.1285	0.011
|22|GO:0001816|cytokine production|	2	94	4.1452	0.16
|23|GO:0005515|protein binding|	42	4552	1.2866	0.15
|24|GO:0008219|cell death|	9	626	2.0869	0.056
|25|GO:0016265| death|	9	626	2.0869	0.056
|26|GO:0006950|response to stress|	8	767	1.5053	0.23
|27|GO:0051239|regulation of multicellular organismal process|	4	241	2.6416	0.11
|28|GO:0005125|cytokine activity|	1	180	0.88632	0.74
|29|GO:0009891|positive regulation of biosynthetic process|	1	58	3.9624	0.36
|30|GO:0005126|hematopoietin cytokine receptor binding|	0	34	0	1

**Table 3:** Protein sequence motifs that are over-represented among known and predicted disease genes. Listed are the top 10 motifs shared in the known disease genes at p<2e-16 (Fisher's exact test), together with their significance in the predicted ones.

InterPro ID	Short Description	InterPro Description	p, for the predicted disease genes
IPR007110	Ig-like	Immunoglobulin-like	0.0004
IPR013151	Immunoglobulin	Immunoglobulin	8.81E-05
IPR003597	Ig_c1	Immunoglobulin C1 type	7.96E-16
IPR003006	Ig_MHC	Immunoglobulin/major histocompatibility complex	1.22E-10
IPR013568	SEFIR	SEFIR	*
IPR000157	TIR	Toll-Interleukin receptor	*
IPR004075	IL1_rcpt_1	Interleukin-1 receptor, type I/Toll precursor	*
IPR001039	MHC_I_alpha_A1A2	MHC class I, alpha chain, alpha1 and alpha2	2.06E-05
IPR001003	MHC_II_alpha_N	MHC class II, alpha chain, N-terminal	2.20E-16
IPR007775	LST1	LST-1	*

* The expected number of genes out of the 68 that share the motif is far below 1, <0.25. The actual number is 0. Not enough power for statistical analysis.

## Abbreviations

T1D: Type 1 Diabetes
HT: High Throughput
PPI: Protein-Protein Interaction
HPRD: Human Protein Reference Database
CC: clustering coefficient
KS-test: Kolmogorov-Smirnov test
